# Relationship between parental physical activity and adolescents' physical activity: the mediating role of family physical activity support

**DOI:** 10.3389/fpubh.2026.1820985

**Published:** 2026-05-14

**Authors:** Fusheng Liang, Hong Ding, Pengfei Gong, Lei Zhang, Laiguo Han

**Affiliations:** 1 School of Physical Education, Huangshan University, Huangshan, Anhui, China; 2 School of Physical Education and Health, East China Normal University, Shanghai, China; 3 College of Humanities and Health, Bengbu Medical University, Bengbu, China

**Keywords:** adolescents, cross-sectional study, family physical activity support, mediation analysis, parental physical activity

## Abstract

**Background:**

Physical activity (PA) in adolescents plays a critical role in their healthy development. Although parental PA is associated with adolescents' PA, the role of family PA support in this relationship remains unclear. This study aims to examine how family PA support mediates the association between parental PA and adolescent PA.

**Methods:**

A 2023 survey conducted across 15 provinces in China ultimately included 11,940 participants. Multiple linear regression examined the links between parental PA, family PA support, and adolescents' PA, adjusting for potential confounders. A mediation model tested whether family PA support mediated the relationship between parental PA and adolescents' PA. Subgroup analyses explored heterogeneity across groups.

**Results:**

After adjusting for confounding variables, parental PA was positively associated with adolescents' PA (β = 0.0969, 95% CI: 0.0796 to 0.1140; *p* < 0.001). Family PA support was positively associated with adolescents' PA (β = 0.3346, 95% CI: 0.3176 to 0.3517, *p* < 0.001). Family PA support mediated 31.99% of the total association of parental PA on adolescents' PA, with a mediation effect size of 0.0310, suggesting that higher parental PA was indirectly linked to increased adolescents' PA through greater family PA support.

**Conclusion:**

The study found that parental PA was positively associated with adolescents' PA, while family PA support was positively associated with adolescents' PA, and family PA support mediated the relationship between parental PA and adolescents' PA.

## Introduction

### Background

Adolescent physical activity (PA) includes all voluntary movements that increase energy expenditure and benefit health—from organized sports and fitness training to active transport, unstructured outdoor play, and daily movement (1, 2). Insufficient PA in youth stems from behavioral, contextual, and relational factors: excessive screen time (3), early-established sedentary habits (4), developmental transitions like entering middle school (5), and limited parental or peer support and modeling (6). PA declines sharply in early adolescence—coinciding with reduced parental involvement in physical activities and increased screen-based leisure (7, 8). This is especially concerning given the well-documented risks of low PA: higher rates of overweight/obesity, impaired glucose metabolism and cardiovascular function, and greater psychological distress—including anxiety, low mood, and reduced wellbeing (7, 9). In China, where adolescent sedentariness is widespread, promoting consistent, age-appropriate PA is critical—not only to address current health threats but also to establish lifelong healthy habits. Therefore, identifying actionable, family-based levers is a priority for scalable, evidence-informed public health strategies.

### Theoretical background

Research shows that adolescents with physically active parents are more likely to be active themselves—and that parental activity can offset the typical decline in PA from late childhood into adolescence (10). Concrete parental involvement—such as walking or playing sports together, and providing transportation, equipment, or scheduling support—is consistently linked to higher adolescent participation in both structured and everyday PA (11, 12). This influence unfolds via several interrelated mechanisms: first, through observational learning, where adolescents adopt active habits by mirroring their parents' routines (13); second, through the broader family ecology—including shared expectations around health behaviors, household activity norms, and resource availability—all of which are shaped by parental practices and socioeconomic circumstances (14, 15). In essence, when parents are physically active, they implicitly communicate that movement is a normal, valued, and rewarding part of daily life. Over time, repeated exposure to such cues helps adolescents internalize positive attitudes toward PA, reinforcing their confidence, intention, and sustained engagement (2, 16).

In China's competitive academic context—where extracurricular time is scarce and lifestyle changes are rapid—parental PA serves as a vital protective factor. The one-child policy has intensified academic pressure from the Gaokao, often causing families to prioritize study over physical activity, which may weaken the translation of parental PA into supportive behaviors. Cross-cultural studies confirm this pattern: parents who prioritize PA consistently foster home environments that encourage, enable, and normalize movement for their children (8, 17).

Parental PA serves two key roles: it models active behavior for adolescents and shapes the family's overall support for movement. Family PA support includes concrete parental actions—such as providing transportation to activities, offering verbal encouragement, engaging in shared PA, and integrating movement into daily routines (6, 12). Research consistently shows that parents who are regularly active are more likely to provide this support. For example, longitudinal data found that adolescents with parents maintaining consistent PA received progressively higher levels of both practical (e.g., access facilitation) and emotional (e.g., praise and encouragement) support over time (8). Similarly, a nationally representative Danish study linked parental PA to stronger logistical support, co-participation, and behavioral modeling—all associated with greater adolescent PA involvement (18). Qualitative evidence adds nuance: active parents often treat PA as a family priority, proactively structuring home life around movement—such as by limiting screen time or scheduling regular outdoor family outings (2, 19). Together, these findings reveal a reciprocal relationship: parental PA doesn't just coincide with family support—it actively sustains it through intentional strategies and everyday transmission of health values and habits.

Inadequate family support for PA and weak social reinforcement are consistently linked to lower adolescent PA engagement and poorer behavioral trajectories (1, 12). In contrast, families that encourage movement, participate alongside their children, and provide affirming feedback foster both stronger motivation and higher PA levels in youth (2, 8, 11). These benefits likely stem from increased self-efficacy—the belief in one's ability to be active—and reduced perception of barriers, core constructs in social cognitive theory (12, 18, 20). Longitudinal and qualitative studies further show that parents who are regularly active are more likely to sustain practical support (e.g., scheduling and transportation), emotional backing, and everyday integration of movement into family life (6, 19). Although prior research has separately examined parental PA and family support as associates of youth activity (6, 12), no study to our knowledge has shown whether family PA support can serve as an explanatory mechanism linking parental PA to adolescent PA, especially in China. Drawing on this gap and the theoretical and empirical foundations outlined above, we propose that family PA support partially accounts for the association between parental PA and adolescent PA.

Thus, parental modeling (behavioral), family PA support (environmental), and adolescent self-efficacy (personal) are interconnected: active parents not only demonstrate PA behaviorally but also foster a supportive environment that strengthens adolescents' confidence to be active.

### The present study

Accordingly, this study tests whether family PA support mediates the association between parental PA and adolescent PA. We hypothesize that parental PA is positively associated with adolescent PA (H1), that family PA support is positively associated with adolescent PA (H2), and that family PA support mediates this relationship (H3). The original contributions are threefold: first large-scale national test of this mediation in China; quantification of the mediation proportion; and identification of relevant moderating factors. These findings advance social cognitive theory for family-based PA promotion.

## Study methodology

### Data collection

**Figure 1:**
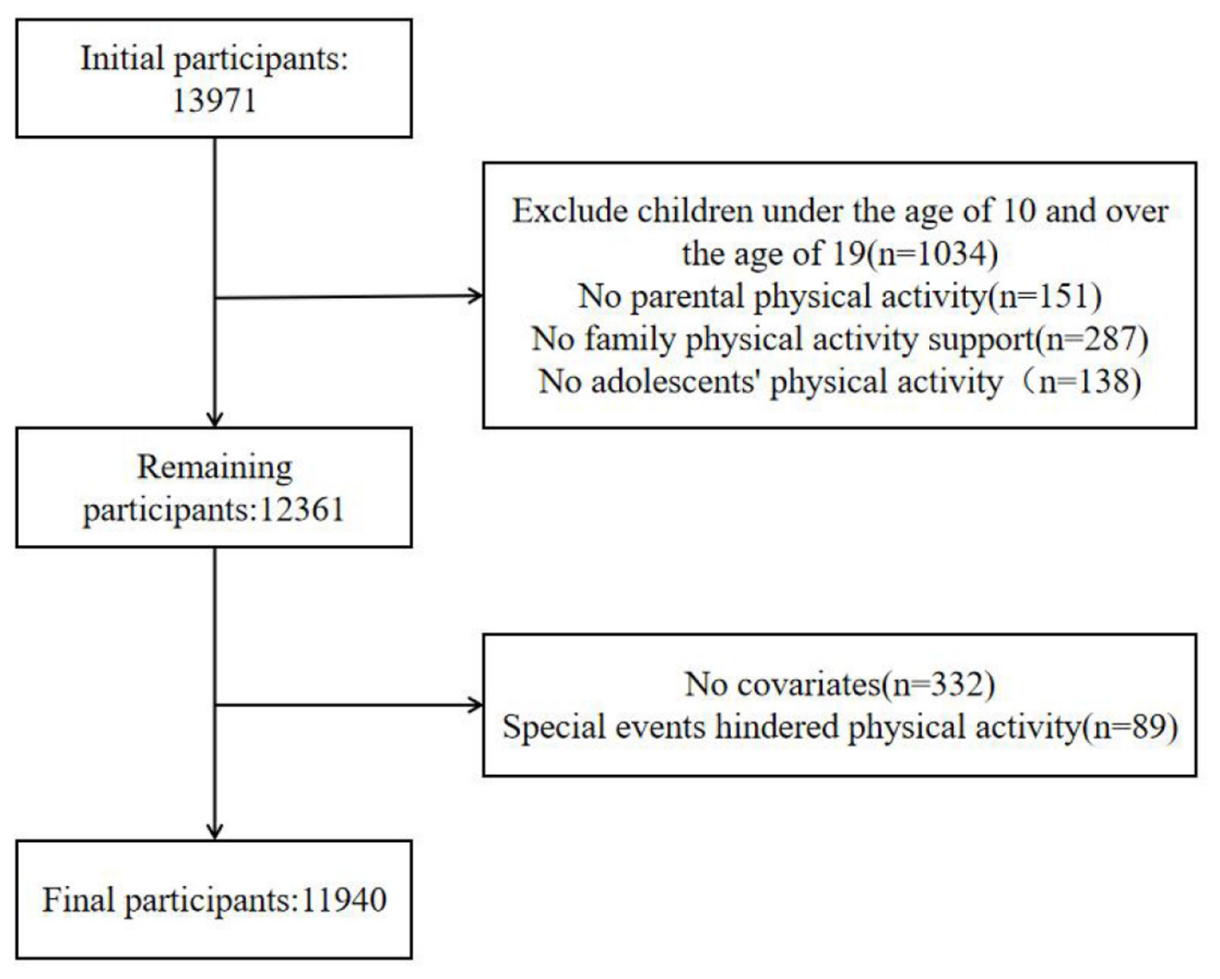
Flowchart of the participants selection process.

This study employed a cross-sectional survey design, implemented as part of the China Children and Adolescents Physical Education and Health Promotion Action—School Construction Project led by East China Normal University. Data were gathered between April and September 2023 across 15 provinces, autonomous regions, and municipalities directly under the central government. The initial sample comprised 13,971 students aged 10–19 years. To ensure analytical validity, we applied a stepwise exclusion protocol: participants outside the target age range (*n* = 1,034), those missing data on parental PA (*n* = 151), family PA support (*n* = 287), adolescent PA (*n* = 138), essential covariates (*n* = 332), or reporting acute physical limitations (e.g., injury or illness temporarily preventing activity; *n* = 89) were sequentially removed. After applying all criteria, the final analytic sample consisted of 11,940 adolescents with fully complete data. The full exclusion pathway is detailed in Figure 1.

### Assessments

#### Adolescents' physical activity

PA of adolescents was measured in 2023 through the Physical Activity Questionnaire-Chinese version (PAQ-CN) (21, 22). PAQ-CN is a self-administered, retrospective physical activity level assessment tool specifically designed for children and adolescents. This questionnaire uses a 4-point Likert scale and its main part consists of 9 items. The total score, after standardization calculation, can reflect the overall physical activity level of the subjects in the past week. The scoring method is divided into 5 steps.

(1) PA1 (Regular PA): It consists of 21 sub-items, covering common PA for children and adolescents such as rope skipping, roller skating, dancing, swimming, and basketball. The participants are required to report the frequency of their participation in each activity over the past week (using a 5-point Likert scale: 1 = never participated, 5 = 7 or more times per week). The final score is the arithmetic mean of the 21 items, which is used to represent the overall participation level of regular PA of the individual.

(2) PA2 to PA8 respectively assess the participants' PA participation levels in seven typical scenarios: physical education classes, breaks, lunch breaks, outside school, evenings, weekends, and holidays. Each question is scored using a 5-point Likert scale (1 = lowest activity level, i.e., almost no PA; 5 = highest activity level, i.e., frequent continuous and moderate to high-intensity activities). The raw scores of each question are directly used as item scores for subsequent dimension analysis and total calculation.

(3) PA9 examines the self-reported daily PA frequency over the past 7 days (1 = never active, 5 = very frequent). The score is the average of the 7-day ratings, reflecting the stability and consistency of short-term activity behavior.

(4) PA10 is a screening item used to identify potential confounding factors that may interfere with regular activity patterns, including acute health events (such as severe colds, dysmenorrhea, fractures, etc.) and temporary external interventions (such as participating in competitive games, artistic rehearsals, or being absent due to going out). If any of the above situations are reported in PA10, the total score of PAQ-CN will not be calculated, and the individual will be excluded from the valid analysis sample to ensure the representativeness and internal validity of the measurement results for the individual's usual PA level.

(5) Calculation and classification standards for the PAQ-CN total score: PA1 to PA9, a total of 9 items, each scored using a 5-point Likert scale (1 to 5 points). The total score is obtained by calculating the arithmetic mean of the 9 raw scores, with the result rounded to two decimal places, forming a continuous variable to represent the quantified level of the individual's PA. This indicator has dual analytical value: first, as a continuous variable, it can be used to describe the overall distribution characteristics and inter-group differences in the PA levels of children and adolescents; second, based on the clinical cut-off points established in previous validation studies, the average score is divided into three categorical variables: low PA level ( ≤ 2.00), moderate PA level (>2.00 and ≤ 3.00), and high PA level (>3.00). This three-level classification has been proven to have good discriminant validity and epidemiological applicability in multiple cross-cultural application studies, which is helpful in identifying risk groups and formulating stratified strategies in public health interventions (23–25).

#### Parental physical activity

Parental PA data were collected in 2023 using a validated short-form version of the International Physical Activity Questionnaire (IPAQ), a standardized instrument designed to quantify time spent in vigorous-, moderate-, and light-intensity activities across multiple domains (26). From this, two composite indicators were constructed: ^*^leisure-time physical activity^*^ (LPA), reflecting purposeful exercise and recreational movement, and ^*^total physical activity^*^ (TPA), which integrates activity from all contexts—including leisure, occupation, transportation, and household tasks.

For each intensity level, participants reported both weekly frequency and typical daily duration. Daily duration was coded into four ordinal categories: 1 = ≤ 30 min, 2 = 31–120 min, 3 = 121–240 min, and 4 = ≥240 min. Weekly exposure was estimated by multiplying frequency by the assigned duration index. These values were then converted into metabolic equivalent of task (MET)-weighted scores using established IPAQ scoring protocols:


$$\begin{array}{l} \text{LPA MET score }=\;\left(8.0\;\times \text{ weekly vigorous LPA}\right)\;+\\ \;\left(4.0\;\times \text{ weekly moderate LPA}\right)\;+\\ \;\left(3.3\;\times \text{ weekly walkingLPA}\right);\\ \text{TPA MET score }=\;\left(8.0\;\times \text{ weekly vigorous TPA}\right)\;+\\ \;\left(4.0\;\times \text{ weekly moderate TPA}\right)\;+\\ \;\left(3.3\;\times \text{ weekly walkingTPA}\right). \end{array}$$

To facilitate analysis, both LPA and TPA were first dichotomized: “inactive” (MET score = 0) vs. “active” (MET score > 0). Among active parents, intensity levels were further stratified into low, moderate, and high tertiles based on their respective MET distributions—ensuring data-driven, population-specific categorization. To reduce response bias—particularly social desirability—we implemented strict anonymity throughout questionnaire administration, including unlinked data collection and confidential handling procedures.

#### Family physical activity support

Family PA support data were gathered in 2023 using the Chinese version of the Activity Support Scale for Multiple Groups (ACTS-CN), a validated, adolescent-reported instrument designed to capture parental influence on youth PA through both behavioral and attitudinal dimensions (26). Adolescents completed the scale independently, rating perceived support from mothers and fathers separately—each assessed via a parallel 9-item module.

Each module comprises two theoretically distinct subscales:

1. Supportive behaviors and attitudes (6 items): Positively worded statements reflecting encouragement, co-participation, logistical facilitation (e.g., arranging transport or access to facilities), reminders, active supervision, and endorsement of structured activities such as sports clubs or training programs. Responses follow a 4-point Likert scale (1 = “strongly disagree”, 4 = “strongly agree”), with raw scores retained.
2. Behavioral restrictions (3 items): Negatively framed items assessing perceived limits on sedentary screen time (e.g., unrestricted TV viewing, computer use, or gaming). To maintain directional consistency, these items were reverse-scored using the formula 5 – response value, so that higher scores consistently reflect greater supportive regulation (e.g., “strongly disagree” with unlimited screen use = 4 points; “strongly agree” = 1 point).

The total ACTS-CN score for each parent is the sum of all nine item scores (six supportive + three reverse-scored restriction items), yielding a composite index ranging from 9 to 36 per parent. Higher totals indicate stronger overall family PA support. To mitigate social desirability bias, the survey was administered anonymously—without identifiers at any stage—and confidentiality was rigorously maintained during instrument development, distribution, and data processing.

### Validity and reliability of the questionnaire

Reliability and validity of all measurement instruments were rigorously evaluated. As summarized in Table 1, internal consistency was assessed using Cronbach's alpha: parental PA (α = 0.611), family PA support (α = 0.840), and adolescent PA (α = 0.827). All coefficients met or exceeded the conventional threshold of 0.6, supporting acceptable reliability.

**Table 1:** Reliability analysis.

Variables	Number of items	Cronbach's α
Parental physical activity	11	0.611
Family physical activity support	18	0.840
Adolescents' physical activity	9	0.827

Construct validity was examined via exploratory factor analysis prerequisites: the Kaiser–Meyer–Olkin (KMO) measure of sampling adequacy and Bartlett's test of sphericity (Table 2). KMO values were 0.671 for parental PA, 0.889 for family PA support, and 0.899 for adolescent PA—all above the recommended minimum of 0.6, indicating sufficient variable correlations for factor analysis. Furthermore, Bartlett's test yielded statistically significant results (all *p* < 0.001), confirming that the correlation matrices were sufficiently intercorrelated to justify scale-level interpretation. Collectively, these findings affirm the psychometric soundness of all three instruments for use in this study.

**Table 2:** Validity analysis.

Variable	Validity		
Parental physical activity	KMO		0.671
	Bartlett's sphericity test	χ^2^	15,973.037
*df*		55
*p*		< 0.001
Family physical activity support	KMO		0.889
	Bartlett's sphericity test	χ^2^	168,178.452
*df*		153
*p*		< 0.001
Adolescents' physical activity	KMO		0.899
	Bartlett's sphericity test	χ^2^	40,701.264
*df*		36
*p*		< 0.001

KMO, Kaiser-Meyer-Olkin; χ^2^, Approx. Chi-Square; df, Degrees of Freedom.

### Confirmatory factor analysis

Confirmatory factor analysis was performed using AMOS 24.0, and the model demonstrated acceptable fit indices: the ratio of chi-square to degrees of freedom (χ^2^/df) was 2.778, the root mean square error of approximation (RMSEA) was 0.076, the adjusted goodness-of-fit index (AGFI) was 0.932, the goodness-of-fit index (GFI) was 0.954, the comparative fit index (CFI) was 0.912, the incremental fit index (IFI) was 0.894, and the normed fit index (NFI) was 0.881, and the standardized root mean square residual value (SRMR) was 0.042. These values indicate that the hypothesized model adequately represents the underlying structure of the observed variables and their relationships with the latent constructs.

As presented in Table 3, all standardized factor loadings for the three latent variables—parental physical activity, family physical activity support, and adolescent physical activity—exceeded 0.5, suggesting that each item effectively represented its corresponding construct. Convergent validity was supported, with average variance extracted (AVE) values all above 0.5 and composite reliability (CR) values exceeding 0.7, indicating satisfactory internal consistency.

**Table 3:** Model regression coefficients.

Path	Non-standardized regression coefficients	Standardized regression coefficient	AVE	CR	*p*
Parental PA → PPA1	1.000	0.726	0.517	0.922	
Parental PA → PPA2	1.455	0.761			< 0.001
Parental PA → PPA3	1.001	0.758			< 0.001
Parental PA → PPA4	0.516	0.702			< 0.001
Parental PA → PPA5	0.679	0.798			< 0.001
Parental PA → PPA6	0.730	0.710			< 0.001
Parental PA → PPA7	0.689	0.706			< 0.001
Parental PA → PPA8	0.580	0.683			< 0.001
Parental PA → PPA9	0.510	0.695			< 0.001
Parental PA → PPA10	0.572	0.672			< 0.001
Parental PA → PPA11	0.616	0.689			< 0.001
Family PA support → Mother1	1.000	0.794	0.604	0.965	
Family PA support → Mother2	1.130	0.807			< 0.001
Family PA support → Mother3	1.107	0.802			< 0.001
Family PA support → Mother4	1.210	0.819			< 0.001
Family PA support → Mother5	1.081	0.771			< 0.001
Family PA support → Mother6	1.110	0.800			< 0.001
Family PA support → Mother7	0.555	0.695			< 0.001
Family PA support → Mother8	0.569	0.698			< 0.001
Family PA support → Mother9	0.428	0.702			< 0.001
Family PA support → Father1	1.300	0.820			< 0.001
Family PA support → Father2	1.487	0.848			< 0.001
Family PA support → Father3	1.493	0.843			< 0.001
Family PA support → Father4	1.489	0.820			< 0.001
Family PA support → Father5	1.270	0.792			< 0.001
Family PA support → Father6	1.371	0.818			< 0.001
Family PA support → Father7	0.406	0.705			< 0.001
Family PA support → Father8	0.416	0.708			< 0.001
Family PA support → Father9	0.462	0.712			< 0.001
Adolescents PA → APA1	1.000	0.788	0.669	0.948	
Adolescents PA → APA2	1.158	0.769			< 0.001
Adolescents PA → APA3	0.646	0.722			< 0.001
Adolescents PA → APA4	0.552	0.715			< 0.001
Adolescents PA → APA5	2.365	0.852			< 0.001
Adolescents PA → APA6	2.378	0.859			< 0.001
Adolescents PA → APA7	2.273	0.874			< 0.001
Adolescents PA → APA8	2.173	0.876			< 0.001
Adolescents PA → APA9	1.964	0.882			< 0.001

Table 4 shows that the absolute correlation coefficients among parental PA, family PA support, and adolescent PA were all lower than the square roots of their respective AVE values. These findings confirm adequate discriminant validity, indicating that while the constructs are interrelated, they remain empirically distinct.

**Table 4:** Discriminant validity.

Variables	Parental PA	Family PA support	Adolescents PA
Parental PA	0.719		
Family PA support	0.084^***^	0.777	
Adolescents PA	0.119^***^	0.417^***^	0.818

p^***^ < 0.001, oblique diagonal numbers are AVE square roots.

As shown in Table 5, the Heterotrait-Monotrait Ratio of Correlations (HTMT) values for each pair of constructs are within a reasonable range and well below the critical threshold of 0.85, indicating good discriminant validity among the factors.

**Table 5:** Heterogeneous to elemental ratio results.

Variables	Parental PA	Family PA support	Adolescents PA
Parental PA	-		
Family PA support	0.109	-	
Adolescents PA	0.137	0.407	-

### Control variables

Baseline covariates included adolescent sex (male/female), parental highest educational attainment (categorized as: no formal schooling, primary education, junior secondary education, senior secondary/vocational or junior college/undergraduate degree, and postgraduate degree or higher), adolescent's current educational stage (primary school, junior high school, or senior high school), and parental body mass index (BMI). Parental height and weight were measured using standardized anthropometric protocols, and BMI was computed as weight (kg) divided by height squared (m^2^). Following WHO classification criteria, parental BMI was classified into four mutually exclusive groups: underweight (< 18.5 kg/m^2^), normal weight (18.5–24.9 kg/m^2^), overweight (25.0–29.9 kg/m^2^), and obesity (≥30.0 kg/m^2^).

### Statistical methods

All statistical analyses for this cross-sectional study were conducted using R software (version 4.3.3). Sample characteristics are summarized as means ± standard deviations for normally distributed continuous variables and as frequencies (percentages) for categorical variables. The Shapiro–Wilk test was applied to assess normality; non-normally distributed continuous variables—including parental PA—were natural log-transformed to improve model assumptions. Bivariate associations among key constructs were evaluated using Spearman's rank correlation coefficients due to non-normality or ordinal scaling. To estimate the magnitude and direction of relationships, multiple linear regression models were fitted with adolescents' PA as the outcome variable. Parental PA and family PA support were entered as primary predictors, and results are reported as standardized β coefficients to facilitate comparison across scales. Two modeling strategies were employed: (1) a minimally adjusted model (unadjusted for covariates), and (2) a fully adjusted model controlling for adolescent grade, sex, parental BMI category, and parental educational attainment. Subgroup analyses were conducted across four pre-specified strata to explore effect modification. To account for multiple testing, the significance threshold was adjusted via Bonferroni correction: α = 0.05 / 4 = 0.0125; thus, only associations with p < 0.0125 were deemed statistically robust in subgroup comparisons. Finally, mediation analysis was performed following the Baron and Kenny framework to assess whether family PA support mediated the association between parental PA and adolescent PA. The indirect effect was estimated using bootstrapping (1,000 resamples), and its significance was determined by whether the 95% confidence interval excluded zero. The proportion mediated—the ratio of the indirect effect to the total effect—was calculated to quantify mediation strength. A two-sided *p* < 0.05 was considered statistically significant for all primary analyses.

## Result

### Characteristics of the study participants

Table 6 summarizes the sociodemographic and anthropometric characteristics of the 11,940 participants. Adolescents' physical activity (PA) scores varied significantly by grade level (*p* < 0.001) and sex (*p* < 0.001), but not by parental BMI (*p* = 0.058). Specifically, PA levels declined progressively across educational stages: students in grades 4–6 reported the highest mean scores, followed by those in grades 7–9, with the lowest values observed among grades 10–12. Males consistently scored higher than females. Moreover, parental education level was significantly associated with adolescent PA (*p* = 0.001), with adolescents whose parents held a bachelor's degree achieving significantly higher PA scores than those whose parents completed only junior secondary education.

**Table 6:** Participants' Basic Characteristics (*N* = 11,940).

Variable		Parental physical activity	Family physical activity support	Adolescents' physical activity
Grade	①Grades 4–6	977.98 ± 878.39	51.83 ± 7.72	2.72 ± 0.73
	②Grades 7–9	1,024.82 ± 921.13	48.44 ± 7.97	2.60 ± 0.75
	③Grades 10–12	1,031.37 ± 890.78	46.57 ± 7.74	2.33 ± 0.76
	*F*	4.477	423.347	202.244
	*p*	0.011	< 0.001	< 0.001
	*LSD*	②③>①	①>②>③	①>②>③
Sex	Male	990.64 ± 888.67	49.90 ± 8.13	2.74 ± 0.78
	Female	1,015.84 ± 903.28	49.70 ± 8.03	2.49 ± 0.71
	*t*	−1.537	1.298	18.590
	*p*	0.124	0.194	< 0.001
Parental BMI	①Underweight	924.40 ± 934.13	50.17 ± 8.34	2.66 ± 0.79
	②Normal weight	985.95 ± 862.69	49.96 ± 8.02	2.63 ± 0.75
	③Overweight	1,049.61 ± 930.30	49.70 ± 7.94	2.61 ± 0.78
	④Obesity	1,043.93 ± 966.50	48.92 ± 8.50	2.58 ± 0.74
	*F*	6.375	6.534	2.500
	*p*	< 0.001	< 0.001	0.058
	*Games-Howell*	③>①② ④>①	①②③>④	-
Parental education level	①Uneducated	584.38 ± 730.67	49.64 ± 8.74	2.58 ± 0.99
	②Elementary school	972.76 ± 1,023.02	48.95 ± 7.99	2.56 ± 0.71
	③Junior high school	1,053.17 ± 951.15	48.74 ± 7.77	2.58 ± 0.74
	④High school	1,091.22 ± 934.84	49.86 ± 7.93	2.63 ± 0.75
	⑤Undergraduate	912.37 ± 802.29	50.57 ± 8.30	2.65 ± 0.78
	⑥Master's degree or above	810.97 ± 654.66	51.99 ± 8.77	2.69 ± 0.79
	*F*	21.078	24.580	3.940
	*p*	< 0.001	< 0.001	0.001
	*Games-Howell*	③④>⑤⑥ ④ >①	⑤⑥>②③④	⑤>③

LSD test was used for *post hoc* analysis, while Games-Howell test was used for statistical analysis in other cases.

Family PA support scores also differed significantly by adolescent grade (*p* < 0.001), parental BMI (*p* < 0.001), and parental education (*p* < 0.001), but not by adolescent sex (*p* = 0.194). Support decreased markedly with advancing grade: strongest in grades 4–6, intermediate in grades 7–9, and weakest in grades 10–12. Across parental BMI categories, underweight, normal-weight, and overweight parents provided significantly greater support than obese parents. Regarding education, family PA support was substantially higher when parents held at least a bachelor's or master's degree, compared to those with elementary, junior secondary, or senior secondary/vocational qualifications.

Parental PA levels showed significant variation by child's grade (*p* = 0.011), parental BMI (*p* < 0.001), and parental education (*p* < 0.001), but not by adolescent sex (*p* = 0.124). Parents of adolescents in grades 7–9 and 10–12 were more active than those of grades 4–6 students. Among BMI groups, overweight parents reported the highest PA levels—significantly exceeding those of underweight, normal-weight, and obese parents. In contrast, parental education exhibited an inverse gradient: those with junior or senior secondary education engaged in significantly more PA than both highly educated (master's degree or above) and least-educated (no formal schooling) parents.

#### The relationship between important variables

Table 7 displays the relationships between parental PA, family PA support, and adolescent PA. In the sample of 11,940 participants, a positive association was observed between parental PA and adolescent PA. The unadjusted model showed a significant link, with β = 0.0875 (95% CI: 0.0696 to 0.1050; *p* < 0.001). This association persisted in the fully adjusted model, with a slight increase to β = 0.0969 (95% CI: 0.0796 to 0.1140; *p* < 0.001), suggesting that covariate adjustment had little influence on the relationship.

**Table 7:** Regression analysis of parental physical activity and family physical activity support on adolescents' physical activity.

Variable	*N*	Unadjusted		Adjusted		Tolerance	VIF
		β (95% CI)	*p*	β (95% CI)	*p*		
Parental physical activity	11,940	0.0875 (0.0696 to 0.1050)	< 0.001	0.0969 (0.0796 to 0.1140)	< 0.001	0.993 to 0.999	1.001 to 1.005
Family physical activity support	11,940	0.3588 (0.3420 to 0.3755)	< 0.001	0.3346 (0.3176 to 0.3517)	< 0.001	0.928 to 0.999	1.001 to 1.078

Adjusted for grade, sex, parental BMI, and parental educational level. *N* = number of participants.

Similarly, family PA support was positively associated with adolescent PA in unadjusted analyses (β = 0.3588, 95% CI: 0.3420 to 0.3755; *p* < 0.001). After full adjustment, the effect size decreased marginally to β = 0.3346 (95% CI: 0.3176 to 0.3517; *p* < 0.001), confirming the stability of this association against potential confounding factors.

The tolerance values (ranging from 0.928 to 0.999) and variance inflation factor (VIF) values (ranging from 1.001 to 1.078) were within acceptable thresholds, indicating no substantial multicollinearity among the predictors.

Supplementary Table 1 indicates that adolescent grade significantly moderated the parental PA–adolescent PA relationship (interaction *P* = 0.001). Subgroup analyses in Supplementary Table 2 revealed no statistically significant effect modification for the association between parental PA and family PA support across any subgroup (all interaction *P* > 0.0125), underscoring its consistency. Supplementary Table 3 further identifies adolescent grade (*P* < 0.001), gender (*P* < 0.001), and parental BMI (*P* = 0.010) as significant effect modifiers of the family PA support–adolescent PA association.

#### Family physical activity support mediates the relationship between parental physical activity and adolescents' physical activity

Table 8 displays the associations between baseline parental PA, family PA support, and adolescent PA. Significant positive correlations were observed: parental PA with adolescent PA (r = 0.088, *p* < 0.001) and with family PA support (r = 0.082, *p* < 0.001). Notably, the correlation between family PA support and adolescent PA was substantially stronger (r = 0.359, *p* < 0.001).

**Table 8:** Association analysis among parental physical activity, family physical activity support and adolescents' physical activity.

Variables	Parental physical activity	Family physical activity support	Adolescents' physical activity
Parental physical activity	1		
Family physical activity support	0.082^***^	1	
Adolescents' physical activity	0.088^***^	0.359^***^	1

p^*^ < 0.05, p^**^ < 0.01, p^***^ < 0.001.

**Figure 2:**
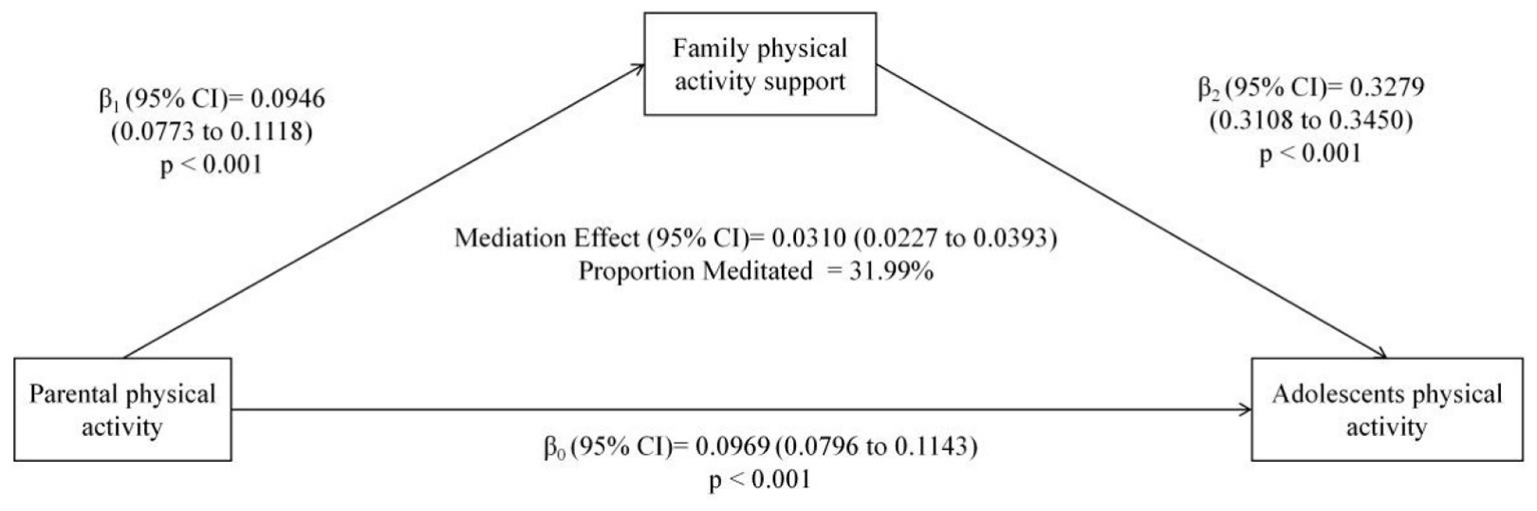
The conceptional framework of the mediation models. β_0_ was the total effect of parental physical activity on adolescents‘ physical activity; β_1_ represents the effect of parental physical activity on family physical activity support; β_2_ represents the effect of family physical activity support on adolescents' physical activity. The mediation effect was computed as the product of “β_1_” and “β_2_” (β_1_ × β_2_), and the mediation proportion was calculated as the ratio of the mediation effect product to total effects [(β_1_ × β_2_)/β_0_].

Bootstrapping analysis indicated a significant total effect of baseline parental PA on adolescent PA (β0= 0.0969, 95% CI: 0.0796 to 0.1143; *p* < 0.001). Family PA support significantly mediated this relationship, with an indirect effect size of 0.0310 (95% CI = 0.0227 to 0.0393). This indirect pathway accounted for 31.99% of the total effect. The mediation model is illustrated in Figure 2.

Table 9 showed that parental PA significantly predicted adolescents' PA (total effect: β = 0.0969, *p* < 0.001; R^2^ = 0.068) and family PA support (path a: β = 0.0946, *p* < 0.001; R^2^ = 0.081). Family PA support strongly predicted adolescents' PA (path b: β = 0.3279, *p* < 0.001), with the full model explaining 16.7% of the variance (R^2^ = 0.167). The direct effect of parental PA remained significant after including the mediator (β = 0.0659, *p* < 0.001).

**Table 9:** Mediation analysis results (*N* = 11,940).

Outcome	Predictor(s)	β	t	R^2^	F	Tolerance	VIF
Adolescents' PA	Parental PA (total effect)	0.0969	10.938^***^	0.068	173.364^***^	0.995	1.005
	Grade	−0.1709	−19.281^***^			0.994	1.006
	Gender	−0.1638	−18.518^***^			0.999	1.001
	Parental BMI	−0.0138	−1.554			0.993	1.007
	Parental education	0.0385	4.338^***^			0.993	1.007
Family PA support	Parental PA (path a)	0.0946	10.749^***^	0.081	210.968^***^	0.995	1.005
	Grade	−0.2513	−28.555^***^			0.994	1.006
	Gender	−0.0060	−0.687			0.999	1.001
	Parental BMI	−0.0222	−2.524^*^			0.993	1.007
	Parental education	0.0911	10.346^***^			0.993	1.007
Adolescents' PA	Parental PA (direct effect, c')	0.0659	7.829^***^	0.167	397.339^***^	0.985	1.015
	Family PA support (path b)	0.3279	37.611^***^			0.919	1.088
	Grade	−0.0885	−10.217^***^			0.931	1.075
	Gender	−0.1618	−19.347^***^			0.999	1.001
	Parental BMI	−0.0065	−0.775			0.993	1.007
	Parental education	0.0086	1.021			0.984	1.016

All models adjusted for grade, gender, parental BMI, and parental educational level. PA, physical activity. ^***^p < 0.001, ^*^p < 0.05.

Across all regression models, tolerance values ranged from 0.919 to 0.999 and VIF values from 1.001 to 1.088, all well within conventional thresholds, indicating no serious multicollinearity among the predictors.

## Discussion

This nationwide cross-sectional study, carried out in 2023 across 15 provinces and provincial-level municipalities in China, enrolled 11,940 adolescents. It examined how parental PA and family PA support relate to adolescent PA levels. Findings confirmed positive associations between both parental PA and family PA support with adolescent PA. Importantly, family PA support was found to partially mediate the parental–adolescent PA link—providing empirical support for the study's primary hypothesis.

Existing literature indicates that parental PA demonstrates significant intergenerational transmission effects on adolescent health behaviors, with enhanced youth participation in PA representing a primary manifestation (5). Consistent with established developmental frameworks (10), our investigation confirmed a positive relationship between parental and adolescent PA after controlling for confounding variables. The underlying mechanisms operate through a multi-level process: initially, parents function as behavioral exemplars, whereby adolescents observing regular parental engagement in PA tend to internalize active lifestyle values and develop stronger self-efficacy beliefs, consequently increasing their own activity involvement (2). Additionally, active parents typically establish supportive household environments through practical assistance (e.g., transportation provisions, equipment acquisition), shared activities, and movement-oriented family routines. These environmental modifications effectively mitigate both practical and perceptual barriers to adolescent PA engagement (6). The synergistic combination of parental modeling and concrete support mechanisms effectively translates parental behavioral patterns into sustained adolescent participation, highlighting parental PA as a foundational element for family-focused intervention strategies.

This study identifies a significant positive link between parental PA and family PA support. Greater parental PA is associated with a more enabling family environment, driven by synergistic behavioral, cognitive, and structural pathways. First, active parents are more inclined to embed PA into the family's shared values and everyday life—such as organizing regular weekend hikes, family cycling outings, or co-participating in team sports—thereby reframing PA not as an individual pursuit but as a collective family norm (19). Second, they deliver instrumental support: acquiring sports gear, enrolling adolescents in organized programs, and facilitating access through transportation and scheduling. These concrete actions lower logistical and financial barriers to participation (6). Third, they supply consistent emotional reinforcement—offering praise, celebrating effort, and visibly expressing enjoyment of movement—which strengthens adolescents' confidence and motivation to engage (19). Notably, this supportive capacity is further strengthened among parents with higher educational attainment, indicating that socioeconomic advantages can augment the translation of personal activity habits into effective family-level facilitation (13). Collectively, these mechanisms demonstrate how parental PA functions as a catalyst for a supportive family ecology—one that actively fosters adolescent participation, even in the absence of direct imitation or behavioral mimicry.

Family PA support has been consistently identified as a critical factor influencing adolescent health behavior patterns (18). Our findings corroborate this association, demonstrating a significant positive relationship between family PA support and adolescent PA engagement, with family support mediating nearly one-third (31.99%) of the total effect between parental and adolescent PA. According to social cognitive theory, this mediation operates through multiple concurrent pathways. Instrumental assistance—including coordinating exercise schedules, supplying necessary equipment, and facilitating transportation—effectively addresses practical constraints related to time availability and material resources. This practical foundation strengthens perceived behavioral control among adolescents and reinforces the viability and value of regular physical activity (1). Concurrently, affective support—manifested through motivational encouragement, shared participation, and positive feedback—helps mitigate psychological obstacles such as performance anxiety or social discomfort. These affirmative interactions build self-efficacy and promote willingness to attempt new physical activities (7). Adolescents receiving substantial family support also exhibit fewer depressive symptoms, suggesting a potential emotional pathway that reinforces sustained participation (27). Furthermore, the integration of PA into regular family routines—such as daily walks or weekly cycling excursions—helps normalize active behaviors. When physical activity becomes embedded in family identity rather than maintained as discrete events, it fosters intrinsic motivation and long-term adherence (18). These interconnected mechanisms demonstrate how family PA support serves as both a structural and motivational bridge, effectively converting parental influence into sustained adolescent engagement through practical enablement and psychological empowerment.

Although family PA support mediated approximately 32% of the total association between parental PA and adolescent PA, a substantial proportion of the effect remains unexplained by this single mediator. This finding suggests that additional pathways likely operate in parallel. First, direct behavioral modeling and observational learning may enable adolescents to acquire active habits by simply watching and replicating their parents' movement patterns, without requiring explicit support behaviors (13). Second, repeated exposure to active parents may enhance adolescents' self-efficacy beliefs regarding PA, as successful modeling increases confidence in one's own ability to overcome barriers and sustain effort (20). Third, active parents often embed movement into daily routines, thereby fostering internalized family health norms and values that make PA an expected, rewarding part of family life rather than a prescribed task (15). Fourth, beyond the family sphere, peer influence and the broader social environment—including friends' activity levels, neighborhood safety, and availability of recreational spaces—can amplify or dampen the transmission of parental activity patterns (1). Finally, school-based PA opportunities, such as physical education quality and after-school sports programs, may interact with parental activity to shape adolescents' overall PA engagement (5).

The finding that family PA support accounts for 31.99% of the association between parental and adolescent PA carries important public health implications. This substantial mediation effect suggests that parental influence operates primarily through cultivating a supportive family ecosystem rather than through direct behavioral modeling alone. In the Chinese context, where intense academic demands severely limit youth PA time, interventions should prioritize strengthening family support capacities. Community-based programs offering time-management coaching, accessible recreational facilities, and structured parent-child co-activity sessions could help families translate intentions into action. By treating the family as an integrated intervention unit, public health strategies can leverage this modifiable pathway to promote adolescent PA, even when direct parental modeling is constrained by structural or cultural barriers.

Future studies should employ longitudinal designs to establish temporal precedence among parental PA, family PA support, and adolescent PA. Objective measures such as accelerometers would enhance validity and reduce self-report bias. Family-based randomized controlled trials are needed to test causal mediation. Research should also examine key moderators including socioeconomic status, urban-rural residence, and cultural contexts, as well as peer influences that may shape these associations across diverse populations.

## Advantages and limitations

This study possesses several strengths. First, the large-scale sample of 11,940 adolescents from 15 provinces across China enhanced statistical power and generalizability to diverse adolescent populations within the national context. Second, validated instruments were employed, including the IPAQ for parental PA, the ACTS-CN for family PA support, and a reliable scale for adolescent PA, ensuring robust measurement of core constructs. Third, the comprehensive analytical approach incorporated mediation modeling, stratification by key demographic factors, and adjustment for potential confounders, strengthening the robustness of observed associations.

However, several limitations should be acknowledged. First, the cross-sectional design precludes causal inference, as temporal order cannot be established and reverse causality remains possible. Second, all variables were self-reported, introducing potential recall and social desirability bias, with no objective measures such as accelerometers utilized. Third, despite covariate adjustment, residual confounding from unmeasured factors including parental health attitudes, adolescent personality traits, and peer influence may have inflated the mediation effect. Fourth, the exclusion of 2,031 participants due to missing data or special circumstances may have introduced selection bias, as differences existed between included and excluded groups. We recommend that future longitudinal studies assess attrition bias and determine whether missing data mechanisms—such as missing completely at random or missing at random—justify imputation or weighting. Fifth, the parental PA measure showed modest internal consistency (α = 0.611). This is expected given the instrument‘s brevity and multi-domain structure (covering leisure, transport, and occupational activities), which can lower alpha coefficients. Nonetheless, this limitation may have attenuated our effect estimates. Future research should consider using objective PA monitors or longer-form questionnaires to improve reliability. Sixth, our data were hierarchical (students were nested within schools and classes). We did not use multilevel models to account for potential clustering effects, which may violate the independence assumption of ordinary least squares regression. Future studies should consider multilevel mediation models to address this limitation. Finally, key ecological influences such as school environment and peer norms were not assessed, and underlying psychological mechanisms were not directly measured, leaving the precise mediating processes incompletely understood.

## Conclusion

This cross-sectional investigation reveals significant positive relationships between parental PA and adolescent PA, as well as between family PA support and adolescent PA levels. Mediation analysis indicates that family PA support functions as a mediating factor in the parental-adolescent PA relationship. These results illuminate a modifiable pathway through which parental PA behaviors may influence adolescent activity patterns, highlighting potential intervention targets for future longitudinal studies and family-focused PA promotion initiatives.

## Data Availability

The original contributions presented in the study are included in the article/Supplementary material, further inquiries can be directed to the corresponding author.
