# Supplementary material for: Relationship between parental physical activity and adolescents' physical activity: the mediating role of family physical activity support
Source: Front Public Health. 2026 May 14;14:1820985. doi: 10.3389/fpubh.2026.1820985 (PMC13215986; doi:10.3389/fpubh.2026.1820985)
Supplement: Supplementary file 1 [file Table_1.docx]

Supplementary Table 1. Subgroup analysis of the association between parental physical activity and adolescents' physical activity

| **Variable** | ***N*** | **Adolescents' physical activity** | ***Coefficient (95% CI)*** | ***P for interaction*** |
| --- | --- | --- | --- | --- |
| Grade |  |  |  | 0.001 |
| Grades 4-6 | 5846 | 2.72±0.73 | 2.26×10^-5^(1.83 ×10^-5^, 2.70 ×10^-5^) |  |
| Grades 7-9 | 4205 | 2.60±0.75 | 1.19×10^-5^(6.80×10^-6^, 1.71×10^-5^) |  |
| Grades 10-12 | 1889 | 2.33±0.76 | 9.20×10^-6^(1.10×10^-6^, 1.73×10^-6^) |  |
| Sex |  |  |  | 0.043 |
| Male | 6121 | 2.74±0.78 | 1.99×10^-5^(1.53×10^-5^, 2.44×10^-5^) |  |
| Female | 5819 | 2.49±0.71 | 1.35×10^-5^(9.30×10^-5^, 1.76×10^-5^) |  |
| Parental BMI |  |  |  | 0.344 |
| Underweight | 814 | 2.66±0.79 | 2.29×10^-5^(1.02×10^-5^, 3.56×10^-5^) |  |
| Normal weight | 7045 | 2.63±0.75 | 1.72×10^-5^(1.31×10^-5^, 2.14×10^-5^) |  |
| Overweight | 2846 | 2.61±0.78 | 1.57×10^-5^(9.2×10^-6^, 2.21×10^-5^) |  |
| Obesity | 1235 | 2.58±0.74 | 9.80×10^-5^(9.00×10^-7^, 1.87×10^-5^) |  |
| Parental education  level |  |  |  | 0.035 |
| Uneducated | 22 | 2.58±0.99 | 7.44×10^-5^(-3.08×10^-5^, 17.95×10^-5^) |  |
| Elementary school | 511 | 2.56±0.71 | 2.68×10^-5^(1.40×10^-5^, 3.95×10^-5^) |  |
| Junior high school | 3411 | 2.58±0.74 | 1.01×10^-5^(4.70×10^-6^, 1.55×10^-5^) |  |
| High school | 3361 | 2.63±0.75 | 1.76×10^-5^(1.20×10^-5^, 2.32×10^-5^) |  |
| Undergraduate | 4400 | 2.65±0.78 | 2.12×10^-5^(1.53×10^-5^, 2.72×10^-5^) |  |
| Master's degree or above | 235 | 2.69±0.79 | 1.90×10^-5^(-1.41×10^-5^, 5.22×10^-5^) |  |
