# Supplementary material for: Relationship between parental physical activity and adolescents' physical activity: the mediating role of family physical activity support
Source: Front Public Health. 2026 May 14;14:1820985. doi: 10.3389/fpubh.2026.1820985 (PMC13215986; doi:10.3389/fpubh.2026.1820985)
Supplement: Supplementary file 2 [file Table_2.docx]

Supplementary Table 2. Subgroup analysis of the association between parental physical activity and family physical activity support

| **Variable** | ***N*** | **Family physical activity support** | ***Coefficient (95% CI)*** | ***P for interaction*** |
| --- | --- | --- | --- | --- |
| Grade |  |  |  | 0.027 |
| Grades 4-6 | 5846 | 51.83±7.72 | 1.90×10^-4^ (1.44×10^-4^, 2.36×10^-4^) |  |
| Grades 7-9 | 4205 | 48.44±7.97 | 1.59×10^-4^ (1.05×10^-4^, 2.14×10^-4^) |  |
| Grades 10-12 | 1889 | 46.57±7.74 | 6.0×10^-5^ (-2.30×10^-5^, 1.42×10^-4^) |  |
| Sex |  |  |  | 0.812 |
| Male | 6121 | 49.90±8.13 | 1.53 ×10^-4^(1.05×10^-4^, 2.01×10^-4^) |  |
| Female | 5819 | 49.70±8.03 | 1.45×10^-4^ (9.80×10^-5^, 1.92×10^-4^) |  |
| Parental BMI |  |  |  | 0.713 |
| Underweight | 814 | 50.17±8.34 | 1.59×10^-4^ (2.50×10^-5^, 2.94×10^-4^) |  |
| Normal weight | 7045 | 49.96±8.02 | 1.47×10^-4^ (1.03×10^-4^, 1.92×10^-4^) |  |
| Overweight | 2846 | 49.70±7.94 | 1.76×10^-4^ (1.11×10^-4^, 2.42×10^-4^) |  |
| Obesity | 1235 | 48.92±8.50 | 1.08×10^-4^ (5.00×10^-6^, 2.10×10^-4^) |  |
| Parental education  level |  |  |  | 0.550 |
| Uneducated | 22 | 49.64±8.74 | 5.92×10^-4^ (-3.49×10^-4^, 1.53×10^-4^) |  |
| Elementary school | 511 | 48.95±7.99 | 2.08×10^-4^ (6.40×10^-5^, 3.52×10^-4^) |  |
| Junior high school | 3411 | 48.74±7.77 | 1.44×10^-4^ (8.80×10^-5^, 2.01×10^-4^) |  |
| High school | 3361 | 49.86±7.93 | 1.52×10^-4^ (9.20×10^-5^, 2.11×10^-4^) |  |
| Undergraduate | 4400 | 50.57±8.30 | 1.85×10^-4^ (1.22×10^-4^, 2.48×10^-4^) |  |
| Master's degree or above | 235 | 51.99±8.77 | 3.86×10^-4^ (2.10×10^-5^, 7.51×10^-4^) |  |
