# Supplementary material for: Relationship between parental physical activity and adolescents' physical activity: the mediating role of family physical activity support
Source: Front Public Health. 2026 May 14;14:1820985. doi: 10.3389/fpubh.2026.1820985 (PMC13215986; doi:10.3389/fpubh.2026.1820985)
Supplement: Supplementary file 3 [file Table_3.docx]

Supplementary Table 3. Subgroup analysis of the association between family physical activity support and adolescents' physical activity

| **Variable** | ***N*** | **Adolescents' physical activity** | ***Coefficient (95% CI)*** | ***P for interaction*** |
| --- | --- | --- | --- | --- |
| Grade |  |  |  | 0.001 |
| Grades 4-6 | 5846 | 2.72±0.73 | 3.40×10^-2^(3.17×10^-2^, 3.63×10^-2^) |  |
| Grades 7-9 | 4205 | 2.60±0.75 | 3.17×10^-2^(2.91×10^-2^, 3.44×10^-2^) |  |
| Grades 10-12 | 1889 | 2.33±0.76 | 2.50×10^-2^(2.07×10^-2^, 2.93×10^-2^) |  |
| Sex |  |  |  | <0.001 |
| Male | 6121 | 2.74±0.78 | 3.04×10^-2^(2.81×10^-2^, 3.26×10^-2^) |  |
| Female | 5819 | 2.49±0.71 | 3.68×10^-2^(3.48×10^-2^, 3.89×10^-2^) |  |
| Parental BMI |  |  |  | 0.010 |
| Underweight | 814 | 2.66±0.79 | 4.04 ×10^-2^(3.45×10^-2^, 4.63×10^-2^) |  |
| Normal weight | 7045 | 2.63±0.75 | 3.21×10^-2^(3.01×10^-2^, 3.42×10^-2^) |  |
| Overweight | 2846 | 2.61±0.78 | 3.65×10^-2^(3.32×10^-2^, 3.99×10^-2^) |  |
| Obesity | 1235 | 2.58±0.74 | 3.11×10^-2^(2.66×10^-2^, 3.57×10^-2^) |  |
| Parental education  level |  |  |  | 0.808 |
| Uneducated | 22 | 2.58±0.99 | 2.06×10^-2^(-2.80×10^-2^, 6.92×10^-2^) |  |
| Elementary school | 511 | 2.56±0.71 | 3.13×10^-2^(2.41×10^-2^, 3.86×10^-2^) |  |
| Junior high school | 3411 | 2.58±0.74 | 3.26×10^-2^(2.96×10^-2^, 3.56×10^-2^) |  |
| High school | 3361 | 2.63±0.75 | 3.50×10^-2^(3.20×10^-2^, 3.79×10^-2^) |  |
| Undergraduate | 4400 | 2.65±0.78 | 3.36×10^-2^(3.10×10^-2^, 3.62×10^-2^) |  |
| Master's degree or above | 235 | 2.69±0.79 | 3.58×10^-2^(2.52×10^-2^, 4.64×10^-2^) |  |
